# Supplementary figures and images for: Seroprevalence of severe fever with thrombocytopenia syndrome using specimens from the Korea National Health & Nutrition Examination Survey
Source: PLoS Negl Trop Dis. 2023 Mar 22;17(3):e0011097. doi: 10.1371/journal.pntd.0011097 (PMC10032665; doi:10.1371/journal.pntd.0011097)

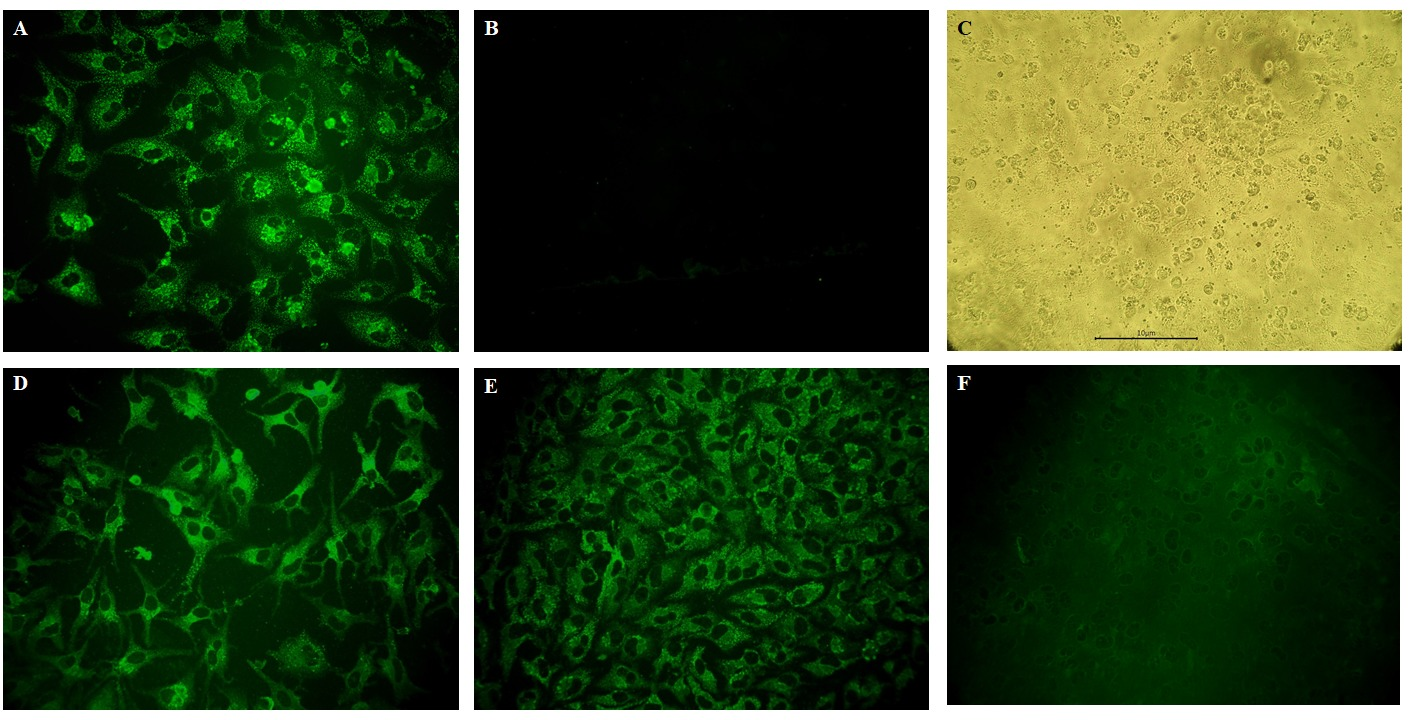

Supplement: S1 Fig — A. IFA image of positive control; B. IFA image of negative control; C. Negative control image showing no staining; D. No.149 serum of the 1st batch (positive, image using serum with 1:32 dilution); E. No.108 serum of the 2nd batch (positive, image using serum with 1:32 dilution); F. No.337 serum of the 2nd batch (negative, image using serum with 1:32 dilution). (TIF) [file pntd.0011097.s001.tif]

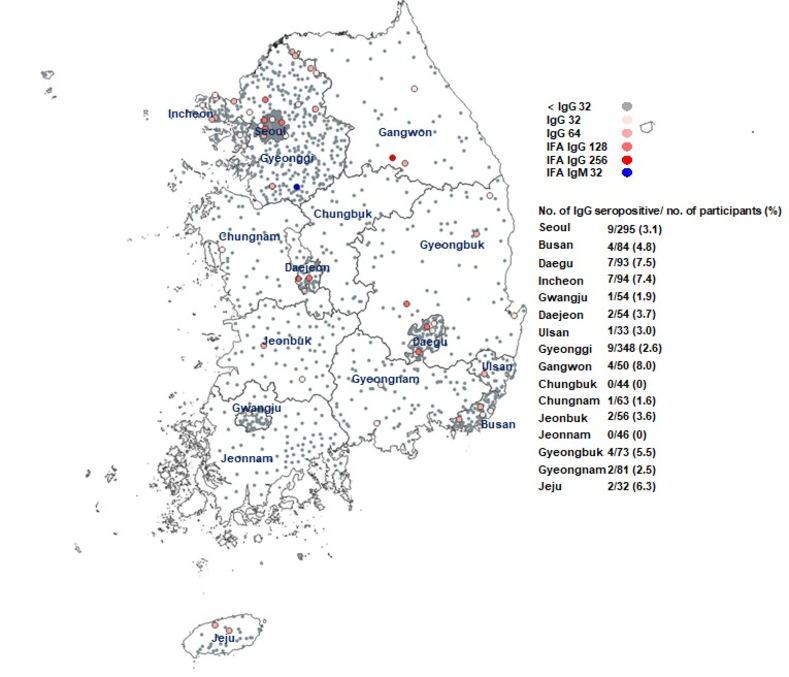

Supplement: S2 Fig — The location of the dot showing the antibody titers in each region was randomly assigned in each region using by QGIS 3.26 program (https://qgis.org/ko/site/) and base map obtained from GEOSERVICE (Administrative Region of South Korea. http://www.gisdeveloper.co.kr/?p=8555#comments). (TIF) [file pntd.0011097.s002.tif]
