# Supplementary material for: Seroprevalence of severe fever with thrombocytopenia syndrome using specimens from the Korea National Health & Nutrition Examination Survey
Source: PLoS Negl Trop Dis. 2023 Mar 22;17(3):e0011097. doi: 10.1371/journal.pntd.0011097 (PMC10032665; doi:10.1371/journal.pntd.0011097)
Supplement: S1 Table — (DOCX) [file pntd.0011097.s003.docx]

S1 Table. The number of participants according to regions in this study, the population of each region, and the ratio of participants to the population (%).

|  | The number of participants in each region out of 1500 participants in this study | | | The population of each region in 2014 | | | The ratio of participants to the population (%) | | |
| --- | --- | --- | --- | --- | --- | --- | --- | --- | --- |
|  | Total no. | Men | Women | Total no. | Men population | Women population | Ratio of total | Ratio of men | Ratio of women |
| Seoul | 295 | 148 | 147 | 10,103,233 | 4,979,768 | 5,123,465 | 0.0029 | 0.0030 | 0.0029 |
| Busan | 84 | 42 | 42 | 3,519,401 | 1,740,417 | 1,778,984 | 0.0024 | 0.0024 | 0.0024 |
| Daegu | 93 | 47 | 46 | 2,493,264 | 1,241,119 | 1,252,145 | 0.0037 | 0.0038 | 0.0037 |
| Incheon | 94 | 47 | 47 | 2,902,608 | 1,459,074 | 1,443,534 | 0.0032 | 0.0032 | 0.0033 |
| Gwangju | 54 | 27 | 27 | 1,475,884 | 731,339 | 744,545 | 0.0037 | 0.0037 | 0.0036 |
| Daejeon | 54 | 27 | 27 | 1,531,809 | 766,497 | 765,312 | 0.0035 | 0.0035 | 0.0035 |
| Ulsan | 33 | 15 | 18 | 1,166,377 | 600,904 | 565,473 | 0.0028 | 0.0025 | 0.0032 |
| Gyeonggi | 348 | 175 | 173 | 12,357,830 | 6,219,813 | 6,138,017 | 0.0028 | 0.0028 | 0.0028 |
| Gangwon | 50 | 25 | 25 | 1,544,442 | 778,007 | 766,435 | 0.0032 | 0.0032 | 0.0033 |
| Chungbuk | 44 | 23 | 21 | 1,578,933 | 796,141 | 782,792 | 0.0028 | 0.0029 | 0.0027 |
| Chungnam | 63 | 31 | 32 | 2,062,273 | 1,045,892 | 1,016,381 | 0.0031 | 0.0030 | 0.0031 |
| Jeonbuk | 56 | 28 | 28 | 1,871,560 | 931,536 | 940,024 | 0.0030 | 0.0030 | 0.0030 |
| Jeonnam | 46 | 22 | 24 | 1,905,780 | 951,975 | 953,805 | 0.0024 | 0.0023 | 0.0025 |
| Gyeongbuk | 73 | 35 | 38 | 2,700,794 | 1,356,182 | 1,344,612 | 0.0027 | 0.0026 | 0.0028 |
| Gyeongnam | 81 | 42 | 39 | 3,350,257 | 1,687,114 | 1,663,143 | 0.0024 | 0.0025 | 0.0023 |
| Jeju | 32 | 17 | 15 | 607,346 | 304,656 | 302,690 | 0.0053 | 0.0056 | 0.0050 |
| Total | 1500 | 751 | 749 | 51171791 | 25590434 | 25581357 |  |  |  |
