# Supplementary material for: Seroprevalence of severe fever with thrombocytopenia syndrome using specimens from the Korea National Health & Nutrition Examination Survey
Source: PLoS Negl Trop Dis. 2023 Mar 22;17(3):e0011097. doi: 10.1371/journal.pntd.0011097 (PMC10032665; doi:10.1371/journal.pntd.0011097)
Supplement: S2 Table — (DOCX) [file pntd.0011097.s004.docx]

S2 Table. The number of participants according to age distribution and regions in this study, the population of each region, and the ratio of participants to the population (%).

|  | The number of participants in each region according to age distribution | | | | | | The population of each region in 2014  according to age distribution | | | | | | The ratio of participants to the population (%)  according to age distribution | | | | | |
| --- | --- | --- | --- | --- | --- | --- | --- | --- | --- | --- | --- | --- | --- | --- | --- | --- | --- | --- |
|  | 10s  (10-19) | 20s  (20-29) | 30s  (30-39) | 40s  (40-49) | 50s  (50-59) | >60s | 10s  (10-19) | 20s  (20-29) | 30s  (30-39) | 40s  (40-49) | 50s  (50-59) | >60s | 10s  (10-19) | 20s  (20-29) | 30s  (30-39) | 40s  (40-49) | 50s  (50-59) | >60s |
| Seoul | 9 | 40 | 54 | 59 | 54 | 79 | 275794 | 360393 | 448313 | 590443 | 571483 | 543415 | 0.0033 | 0.0111 | 0.0120 | 0.0100 | 0.0094 | 0.0145 |
| Busan | 2 | 14 | 13 | 12 | 18 | 25 | 97754 | 113500 | 138698 | 195168 | 207829 | 196372 | 0.0020 | 0.0123 | 0.0094 | 0.0061 | 0.0087 | 0.0127 |
| Daegu | 2 | 12 | 12 | 16 | 20 | 31 | 81376 | 81744 | 97291 | 149508 | 136680 | 117163 | 0.0025 | 0.0147 | 0.0123 | 0.0107 | 0.0146 | 0.0265 |
| Incheon | 2 | 11 | 10 | 20 | 19 | 32 | 92160 | 97344 | 122384 | 175628 | 163915 | 123267 | 0.0022 | 0.0113 | 0.0082 | 0.0114 | 0.0116 | 0.0260 |
| Gwangju | 2 | 7 | 10 | 11 | 11 | 13 | 48087 | 46984 | 61112 | 81090 | 65786 | 52223 | 0.0042 | 0.0149 | 0.0164 | 0.0136 | 0.0167 | 0.0249 |
| Daejeon | 2 | 6 | 6 | 14 | 13 | 13 | 46824 | 50059 | 60918 | 84439 | 75851 | 60723 | 0.0043 | 0.0120 | 0.0098 | 0.0166 | 0.0171 | 0.0214 |
| Ulsan | 2 | 2 | 6 | 8 | 8 | 7 | 30001 | 28266 | 40938 | 59520 | 53794 | 38414 | 0.0067 | 0.0071 | 0.0147 | 0.0134 | 0.0149 | 0.0182 |
| Gyeonggi | 10 | 50 | 58 | 72 | 70 | 88 | 387918 | 380518 | 489776 | 740364 | 659849 | 542110 | 0.0026 | 0.0131 | 0.0118 | 0.0097 | 0.0106 | 0.0162 |
| Gangwon | 2 | 5 | 6 | 11 | 11 | 15 | 49012 | 44793 | 54029 | 90958 | 99823 | 108602 | 0.0041 | 0.0112 | 0.0111 | 0.0121 | 0.0110 | 0.0138 |
| Chungbuk | 1 | 3 | 6 | 8 | 11 | 15 | 48394 | 44900 | 56525 | 89058 | 87013 | 86090 | 0.0021 | 0.0067 | 0.0106 | 0.0090 | 0.0126 | 0.0174 |
| Chungnam | 1 | 5 | 12 | 12 | 9 | 24 | 65656 | 59845 | 77796 | 117752 | 116970 | 130098 | 0.0015 | 0.0084 | 0.0154 | 0.0102 | 0.0077 | 0.0184 |
| Jeonbuk | 2 | 5 | 5 | 10 | 12 | 22 | 62214 | 55398 | 70018 | 105645 | 99473 | 111855 | 0.0032 | 0.0090 | 0.0071 | 0.0095 | 0.0121 | 0.0197 |
| Jeonnam | 2 | 5 | 6 | 10 | 8 | 15 | 67225 | 57733 | 72488 | 112739 | 112074 | 149799 | 0.0030 | 0.0087 | 0.0083 | 0.0089 | 0.0071 | 0.0100 |
| Gyeongbuk | 2 | 5 | 9 | 11 | 14 | 32 | 81119 | 76961 | 101497 | 155297 | 154734 | 169223 | 0.0025 | 0.0065 | 0.0089 | 0.0071 | 0.0090 | 0.0189 |
| Gyeongnam | 2 | 8 | 7 | 18 | 15 | 31 | 103731 | 92863 | 123740 | 193692 | 184029 | 172322 | 0.0019 | 0.0086 | 0.0057 | 0.0093 | 0.0082 | 0.0180 |
| Jeju | 2 | 2 | 5 | 8 | 7 | 8 | 24674 | 19895 | 28034 | 44147 | 37139 | 34761 | 0.0081 | 0.0101 | 0.0178 | 0.0181 | 0.0188 | 0.0230 |
| Total | 45 | 180 | 225 | 300 | 300 | 450 | 1561939 | 1611196 | 2043557 | 2985448 | 2826442 | 2636437 |  |  |  |  |  |  |
