# Supplementary material for: Seroprevalence of severe fever with thrombocytopenia syndrome using specimens from the Korea National Health & Nutrition Examination Survey
Source: PLoS Negl Trop Dis. 2023 Mar 22;17(3):e0011097. doi: 10.1371/journal.pntd.0011097 (PMC10032665; doi:10.1371/journal.pntd.0011097)
Supplement: Attachment — Submitted filename: Response letter_SFTS_2nd revision.docx [file pntd.0011097.s005.docx]

**To the Editor of PLOS Neglected Tropical Diseases**

**Re: Manuscript ID: PNTD-D-22-00247R1
Seroprevalence of severe fever with thrombocytopenia syndrome using specimens from the Korea National Health & Nutrition Examination Survey**

Dear Editor,

First, I would like to thank you on behalf of all of my co-authors for reviewing our manuscript.

Our responses to the comments made by the reviewers are included below in bold.

Thank you for considering our manuscript, and we hope that this draft of our manuscript will be of interest to the readers of “PLOS Neglected Tropical Diseases*”*.

**Reviewer(s)' Comments to Author:**

Thank you for submitting your paper, "Seroprevalence of severe fever with thrombocytopenia syndrome using specimens from the Korea National Health & Nutrition Examination Survey", to PLOS Neglected Tropical Diseases. We have checked over your submission and, before we can assign your manuscript to the Editor, we ask that you first please make the following modifications:

● 1) Please ensure that the CRediT author contributions listed for every co-author are completed accurately and in full. At this stage, "All Authors" require contributions. Please ensure that the full contributions of each author are acknowledged in the ""Add/Edit/Remove Authors"" section of our submission form.

The list of CRediT author contributions may be found here:
https://journals.plos.org/plosntds/s/authorship#loc-author-contributions
**Response> Thank you for your valuable comments. As per your recommendation, we have added the full contributions of each author in the ""Add/Edit/Remove Authors"" section.**

● 2) We have noticed that you have uploaded Supporting Information files, but you have not included a list of legends. Please add a full list of legends for your Supporting Information files after the references list.

**Response> Thank you for your valuable comments. As per your recommendation, we have added “Legends for Supporting Information files” after the references list as follows.**

**⇒**

**Legends for Supporting Information files**

**Supplementary Fig 1. Representative indirect immunofluorescence antibody assay results showing the presence of SFTS-specific IgG antibody using serum samples collected in KNHANES.**

**A. IFA image of positive control; B. IFA image of negative control; C. Negative control image showing no staining; D. No.149 serum of the 1st batch (positive, image using serum with 1:32 dilution); E. No.108 serum of the 2nd batch (positive, image using serum with 1:32 dilution); F. No.337 serum of the 2nd batch (negative, image using serum with 1:32 dilution).**

**Supplementary Fig 2. Geographical distribution according to antibody titers of the 1500 participants by regions collected from the Korea National Health and Nutrition Examination Survey, 2014 to 2015. The location of the dot showing the antibody titers in each region was randomly assigned in each region by QGIS 3.26 program (**[**https://qgis.org/ko/site/**](https://qgis.org/ko/site/)**) using map obtained from GIS Developer site (http://www.gisdeveloper.co.kr/?p=2332).**

**Supplementary Table 1. The number of participants according to regions in this study, the population of each region, and the ratio of participants to the population (%).**

**Supplementary Table 2. The number of participants according to age distribution and regions in this study, the population of each region, and the ratio of participants to the population (%).**

● 3) Thank you for including your ethics statement: " This study was approved by the Institutional Review Board (IRB) of Chosun University Hospital (IRB No. 2017-12-014). Written informed consent was obtained from all participants for the use of blood samples in KNHANES. Of the participants, 50.1%(751) were men and 49.9% (749) were women. Approximately 3.0% (n = 45) of the participants were between 10 and 19 years of age, with 12.0% (n = 180), 15.0% (n = 225),20.0% (n = 300), 20.0% (n = 300), and 30.0% (n = 450) between 20 and 29, 30–39, 40–49,50–59, and > 60 years of age, respectively. Please include a statement that formal consent was obtained(must state whether verbal/written) from the parent/guardian/head of household (In case of participants less than 18yrs of age).

**Response> Thank you for your valuable comments. As per your recommendation, we have added following sentence into Ethics statement.**

**⇒ Written informed consent was obtained from the parent/guardian/head of household in case of participants less than 18 years old.**

● 4) Please amend your detailed Financial Disclosure statement. This is published with the article, therefore should be completed in full sentences and contain the exact wording you wish to be published.
i). State the initials, alongside each funding source, of each author to receive each grant.
ii). State what role the funders took in the study. If the funders had no role in your study, please state: “The funders had no role in study design, data collection and analysis, decision to publish, or preparation of the manuscript.”
iii). If any authors received a salary from any of your funders, please state which authors and which funders.

**Response> Thank you for your valuable comments. As per your recommendation, we have changed it as follows.**

**⇒**

**Financial Disclosure**

**C-MK received grant supported by the Research Program funded by the Korea Disease Control and Prevention Agency (fund code 2017P2300300). The funder had no role in study design, data collection and analysis, decision to publish, or preparation of the manuscript.**

● 5) Some material included in your submission may be copyrighted. According to PLOS’s copyright policy, authors who use figures or other material (e.g., graphics, clipart, maps) from another author or copyright holder must demonstrate or obtain permission to publish this material under the Creative Commons Attribution 4.0 International (CC BY 4.0) License used by PLOS journals. Please closely review the details of PLOS’s copyright requirements here: PLOS Licenses and Copyright. If you need to request permissions from a copyright holder, you may use PLOS's Copyright Content Permission form.

Please respond directly to this email and provide any known details concerning your material's license terms and permissions required for reuse, even if you have not yet obtained copyright permissions or are unsure of your material's copyright compatibility. Once you have responded and addressed all other outstanding technical requirements, you may resubmit your manuscript within Editorial Manager.

Potential Copyright Issues:

[Figure 3, Supplementary Figure 2]: please (a) provide a direct link to the base layer of the map (i.e., the country or region border shape) and ensure this is also included in the figure legend; and (b) provide a link to the terms of use / license information for the base layer image or shapefile. We cannot publish proprietary or copyrighted maps (e.g. Google Maps, Mapquest) and the terms of use for your map base layer must be compatible with our CC BY 4.0 license.

Note: if you created the map in a software program like R or ArcGIS, please locate and indicate the source of the basemap shapefile onto which data has been plotted.

If your map was obtained from a copyrighted source please amend the figure so that the base map used is from an openly available source. Alternatively, please provide explicit written permission from the copyright holder granting you the right to publish the material under our CC BY 4.0 license.

If you are unsure whether you can use a map or not, please do reach out and we will be able to help you. The following websites are good examples of where you can source open access or public domain maps:
* U.S. Geological Survey (USGS) - All maps are in the public domain. (http://www.usgs.gov)
* PlaniGlobe - All maps are published under a Creative Commons license so please cite “PlaniGlobe, http://www.planiglobe.com, CC BY 2.0” in the image credit after the caption. (http://www.planiglobe.com/?lang=enl)
* Natural Earth - All maps are public domain. (http://www.naturalearthdata.com/about/terms-of-use/)

**Response> Thank you for your valuable comments. As per your recommendation, we have added “PlaniGlobe, http://www.planiglobe.com, CC BY 2.0” in Figure 3A image. Moreover, we have added following sentence into new Figure 3A legend.**

**⇒ Figure 3A. Choropleth map showing national SFTS seroprevalence (%) with IgG titer of ≥ 1:32. Map was obtained using “Editable South Korea Map Template For Presentation” (**[**https://www.slideegg.com/powerpoint/south-korea-map-powerpoint-templates**](https://www.slideegg.com/powerpoint/south-korea-map-powerpoint-templates)**).**

**For Supplementary Fig 2, map was obtained from GIS Developer site (**[**http://www.gisdeveloper.co.kr/?p=2332**](http://www.gisdeveloper.co.kr/?p=2332)**) and the location of the dot showing the antibody titers in each region was randomly assigned in each region by QGIS 3.26 program (**[**https://qgis.org/ko/site/**](https://qgis.org/ko/site/)**). Therefore, we have added following sentence into Supplementary Fig 2 legend.**

**⇒ Supplementary Fig 2. Geographical distribution according to antibody titers of the 1500 participants by regions collected from the Korea National Health and Nutrition Examination Survey, 2014 to 2015. The location of the dot showing the antibody titers in each region was randomly assigned in each region by QGIS 3.26 program (**[**https://qgis.org/ko/site/**](https://qgis.org/ko/site/)**) using map obtained from GIS Developer site (http://www.gisdeveloper.co.kr/?p=2332).**

● 6) We have noticed that you have included your Data Availability Statement twice in the submission.
Data Availability Statement 1 :" All relevant data are within the manuscript.
Additional data availability information:
Tick here if the URLs/accession numbers/DOIs will be available only after acceptance of the manuscript for publication so that we can ensure their inclusion before publication".

Data Availability Statement 2: "All relevant data are within the manuscript and its Supporting Information files".

Could you please verify which of these two statements is correct?

**Response> Thank you for your valuable comments. During revision, we have added Supplementary Table 1 and 2 in Supporting Information files. Therefore, Data Availability Statement” has been changed. We have finalized “Data Availability Statement” as follows.**

**All relevant data are within the manuscript and its** **Supporting Information files.**

**To the Editor of PLOS Neglected Tropical Diseases**

**Re:** **PNTD-D-22-00247**

**Seroprevalence of severe fever with thrombocytopenia syndrome using specimens from the Korea National Health & Nutrition Examination Survey**

Dear Editor,

First, I would like to thank you on behalf of all of my co-authors for reviewing our manuscript.

Our responses to the comments made by the reviewers are included below in bold.

Thank you for considering our manuscript, and we hope that this draft of our manuscript will be of interest to the readers of “PLOS Neglected Tropical Diseases*”*.

**Reviewer(s)' Comments to Author:**

**Reviewer #1:**

Reviewer #1: Short summary
The researchers have done an excellent job of estimating the seroprevalence of this disease. It is important to keep surveillance in order given the high CFR of SFTS. The current state of the manuscript needs to be improved before proceeding to publication. The points are described below. Main point is to present the data better, for example a better visualization of the representativeness of the sample and argument why a certain cut-off value was chosen.

**Response> Thank you for your valuable comments. We did our best to respond to the comments of reviewers and prepared response letter and revised manuscript. Please see the following response.**

Major points
● Line 80. Please provide estimates within the different risk groups or was it just one estimate for the whole group? I am curious to know what the seroprevalence is in the highest risk group or how much the seroprevalences variates from region to region. In line 84 it is suggested there are large differences between areas, please provide the numbers.

**Response> Thank you for your valuable comments. As per your recommendation, we have added following sentences into “Introduction” of our revised manuscript.**

**⇒ Fifty (4.1%) of the 1,228 residents of rural areas exhibited an SFTS antibody titer of > 1:32, of which 23 (5.5%) in Boseong, 16 (4.0%) in Hapcheon, and 11 (2.7%) in Dangjin were seropositive for the SFTSV [11]. In addition, in a study conducted on 1,069 serum samples from patients who visited a tertiary hospital in southeastern Korea, 2.1% of the patients were positive for anti-SFTSV antibodies, of which 7.7% of patients from rural areas and 1.9% of patients from urban areas were shown to be seropositive [6].**

**⇒ In SFTSV antibody testing on 2,510 residents in Jiangsu Province, China, 0.44% of the residents tested positive [5]. However, the seropositivity rate for farmers in rural areas of the same province was 1.3%, which was three times higher than that of the general population [6, 7]. In addition, the seroprevalence of SFTSV infections was tested in 1,375 healthy persons from Penglai County, eastern China, and the serum of 3.85% of the general population tested positive for SFTSV antibodies [8]. In contrast, 7.2% of healthy residents in Zhejiang Province, China were positive for SFTSV IgG antibodies [9].**

● Line 110. The cut-off is determines who is positive and who is negative. The titers of 1:32 is based on results from 15 health check-up participants. The importance of the cut-off in this study warrants an elobation about how it was at 1:32 instead of referring to another study.

**Response> Thank you for your valuable comments.**

**We performed IFA according to the indirect IFA method provided by the Korea Disease Control and Prevention Agency (KDCA), and positive cut-off values for IgG and IgM antibody were set as titers ≥ 1:32. The cut-off values were confirmed by using sera from 15 health check-up participants, and it was confirmed that all 15 healthy people were negative at titer of 1:32. Moreover, we used serum from a follow-up patients who tested positive for SFTS RT-PCR as positive control for SFTS IFA.**

**In addition, in our previous study (data not shown) investigating SFTS seroprevalence, we performed SFTS IFA, SFTS indirect ELISA, and plaque reduction neutralization antibody test (PRNT) using serum samples from veterinary-hospital staff from January to May 2021. In seroprevalence tests of 103 veterinary-hospital workers, indirect ELISA, IFA, and PRNT yielded positive results in 4 (3.9%), 3 (2.9%), and 2 (1.9%) participants, respectively. Two (who has past history of SFTS) of the 4 participants who tested positive for SFTS indirect ELISA were positive for PRNT and were also positive for SFTS IFA. Antibody positivity was defined as follows: an IFA IgG titer of ≥1:32, indirect ELISA IgG positivity (OD≥ mean + 3SD), and PRNT positivity (titer≥10). So to speak, when using each cut-off for positivity, SFTS seroprevalence determined by ELISA and IFA was similar. Moreover, using cut-off of ≥1:16 for IFA has a probability showing false-positive.**

● Line 134 / Figure 1. A reason to do this study was to obtain representative estimates of the seroprevalence in South Korea. To underline this aim, it would be good to create a figure, where you show what fraction the number of participants are of the total population in each region. Likewise, would compare the age distribution in your sample with the age distribution in Korea. A good example is Figure in 1 in Backer et al. Eurosurveillance 2021 (Impact of physical distancing measures against COVID-19 on contacts and mixing patterns: repeated cross-sectional surveys, the Netherlands, 2016–17, April 2020 and June 2020). This provides information on the representativeness of your sample of the Korean population.

**Response> Thank you for your valuable comments. This study was to investigate nationwide SFTS antibody positivity rate, including that of 16 cities and provinces.**

**We obtained information on population in each region from KOSIS (Korean statistical information service).**

**The percentage of regional participants in this study is 0.0024~0.0053% of the region population, which is too small to be representative of the Korean population.**

**However, based on your recommendation, we have added following tables (supplementary Table 1 and 2) and following sentence to show number of participants among the total population in each region and age distribution into Result section. We hope that the following tables will help you understand.**

**https://kosis.kr/statHtml/statHtml.do?orgId=101&tblId=DT_1B040A3&checkFlag=N**

**⇒ The number of participants according to age distribution and regions, the population of each region in 2014, and the ratio of participants to the population (%) are shown in Supplementary Tables 1 and 2.**

**Supplementary Table 1****. The number of participants according to regions in this study, the population of each region, and the ratio of participants to the population (%).**

|  | The number of participants in each region out of 1500 participants in this study | | | The population of each region in 2014 | | | The ratio of participants to the population (%) | | |
| --- | --- | --- | --- | --- | --- | --- | --- | --- | --- |
|  | Total no. | Men | Women | Total no. | Men population | Women population | Ratio of total | Ratio of men | Ratio of women |
| Seoul | 295 | 148 | 147 | 10,103,233 | 4,979,768 | 5,123,465 | 0.0029 | 0.0030 | 0.0029 |
| Busan | 84 | 42 | 42 | 3,519,401 | 1,740,417 | 1,778,984 | 0.0024 | 0.0024 | 0.0024 |
| Daegu | 93 | 47 | 46 | 2,493,264 | 1,241,119 | 1,252,145 | 0.0037 | 0.0038 | 0.0037 |
| Incheon | 94 | 47 | 47 | 2,902,608 | 1,459,074 | 1,443,534 | 0.0032 | 0.0032 | 0.0033 |
| Gwangju | 54 | 27 | 27 | 1,475,884 | 731,339 | 744,545 | 0.0037 | 0.0037 | 0.0036 |
| Daejeon | 54 | 27 | 27 | 1,531,809 | 766,497 | 765,312 | 0.0035 | 0.0035 | 0.0035 |
| Ulsan | 33 | 15 | 18 | 1,166,377 | 600,904 | 565,473 | 0.0028 | 0.0025 | 0.0032 |
| Gyeonggi | 348 | 175 | 173 | 12,357,830 | 6,219,813 | 6,138,017 | 0.0028 | 0.0028 | 0.0028 |
| Gangwon | 50 | 25 | 25 | 1,544,442 | 778,007 | 766,435 | 0.0032 | 0.0032 | 0.0033 |
| Chungbuk | 44 | 23 | 21 | 1,578,933 | 796,141 | 782,792 | 0.0028 | 0.0029 | 0.0027 |
| Chungnam | 63 | 31 | 32 | 2,062,273 | 1,045,892 | 1,016,381 | 0.0031 | 0.0030 | 0.0031 |
| Jeonbuk | 56 | 28 | 28 | 1,871,560 | 931,536 | 940,024 | 0.0030 | 0.0030 | 0.0030 |
| Jeonnam | 46 | 22 | 24 | 1,905,780 | 951,975 | 953,805 | 0.0024 | 0.0023 | 0.0025 |
| Gyeongbuk | 73 | 35 | 38 | 2,700,794 | 1,356,182 | 1,344,612 | 0.0027 | 0.0026 | 0.0028 |
| Gyeongnam | 81 | 42 | 39 | 3,350,257 | 1,687,114 | 1,663,143 | 0.0024 | 0.0025 | 0.0023 |
| Jeju | 32 | 17 | 15 | 607,346 | 304,656 | 302,690 | 0.0053 | 0.0056 | 0.0050 |
| Total | 1500 | 751 | 749 | 51171791 | 25590434 | 25581357 |  |  |  |

**Supplementary Table 2. The number of participants according to age distribution and regions in this study, the population of each region, and the ratio of participants to the population (%).**

|  | The number of participants in each region according to age distribution | | | | | | The population of each region in 2014  according to age distribution | | | | | | The ratio of participants to the population (%)  according to age distribution | | | | | |
| --- | --- | --- | --- | --- | --- | --- | --- | --- | --- | --- | --- | --- | --- | --- | --- | --- | --- | --- |
|  | 10s  (10-19) | 20s  (20-29) | 30s  (30-39) | 40s  (40-49) | 50s  (50-59) | >60s | 10s  (10-19) | 20s  (20-29) | 30s  (30-39) | 40s  (40-49) | 50s  (50-59) | >60s | 10s  (10-19) | 20s  (20-29) | 30s  (30-39) | 40s  (40-49) | 50s  (50-59) | >60s |
| Seoul | 9 | 40 | 54 | 59 | 54 | 79 | 275794 | 360393 | 448313 | 590443 | 571483 | 543415 | 0.0033 | 0.0111 | 0.0120 | 0.0100 | 0.0094 | 0.0145 |
| Busan | 2 | 14 | 13 | 12 | 18 | 25 | 97754 | 113500 | 138698 | 195168 | 207829 | 196372 | 0.0020 | 0.0123 | 0.0094 | 0.0061 | 0.0087 | 0.0127 |
| Daegu | 2 | 12 | 12 | 16 | 20 | 31 | 81376 | 81744 | 97291 | 149508 | 136680 | 117163 | 0.0025 | 0.0147 | 0.0123 | 0.0107 | 0.0146 | 0.0265 |
| Incheon | 2 | 11 | 10 | 20 | 19 | 32 | 92160 | 97344 | 122384 | 175628 | 163915 | 123267 | 0.0022 | 0.0113 | 0.0082 | 0.0114 | 0.0116 | 0.0260 |
| Gwangju | 2 | 7 | 10 | 11 | 11 | 13 | 48087 | 46984 | 61112 | 81090 | 65786 | 52223 | 0.0042 | 0.0149 | 0.0164 | 0.0136 | 0.0167 | 0.0249 |
| Daejeon | 2 | 6 | 6 | 14 | 13 | 13 | 46824 | 50059 | 60918 | 84439 | 75851 | 60723 | 0.0043 | 0.0120 | 0.0098 | 0.0166 | 0.0171 | 0.0214 |
| Ulsan | 2 | 2 | 6 | 8 | 8 | 7 | 30001 | 28266 | 40938 | 59520 | 53794 | 38414 | 0.0067 | 0.0071 | 0.0147 | 0.0134 | 0.0149 | 0.0182 |
| Gyeonggi | 10 | 50 | 58 | 72 | 70 | 88 | 387918 | 380518 | 489776 | 740364 | 659849 | 542110 | 0.0026 | 0.0131 | 0.0118 | 0.0097 | 0.0106 | 0.0162 |
| Gangwon | 2 | 5 | 6 | 11 | 11 | 15 | 49012 | 44793 | 54029 | 90958 | 99823 | 108602 | 0.0041 | 0.0112 | 0.0111 | 0.0121 | 0.0110 | 0.0138 |
| Chungbuk | 1 | 3 | 6 | 8 | 11 | 15 | 48394 | 44900 | 56525 | 89058 | 87013 | 86090 | 0.0021 | 0.0067 | 0.0106 | 0.0090 | 0.0126 | 0.0174 |
| Chungnam | 1 | 5 | 12 | 12 | 9 | 24 | 65656 | 59845 | 77796 | 117752 | 116970 | 130098 | 0.0015 | 0.0084 | 0.0154 | 0.0102 | 0.0077 | 0.0184 |
| Jeonbuk | 2 | 5 | 5 | 10 | 12 | 22 | 62214 | 55398 | 70018 | 105645 | 99473 | 111855 | 0.0032 | 0.0090 | 0.0071 | 0.0095 | 0.0121 | 0.0197 |
| Jeonnam | 2 | 5 | 6 | 10 | 8 | 15 | 67225 | 57733 | 72488 | 112739 | 112074 | 149799 | 0.0030 | 0.0087 | 0.0083 | 0.0089 | 0.0071 | 0.0100 |
| Gyeongbuk | 2 | 5 | 9 | 11 | 14 | 32 | 81119 | 76961 | 101497 | 155297 | 154734 | 169223 | 0.0025 | 0.0065 | 0.0089 | 0.0071 | 0.0090 | 0.0189 |
| Gyeongnam | 2 | 8 | 7 | 18 | 15 | 31 | 103731 | 92863 | 123740 | 193692 | 184029 | 172322 | 0.0019 | 0.0086 | 0.0057 | 0.0093 | 0.0082 | 0.0180 |
| Jeju | 2 | 2 | 5 | 8 | 7 | 8 | 24674 | 19895 | 28034 | 44147 | 37139 | 34761 | 0.0081 | 0.0101 | 0.0178 | 0.0181 | 0.0188 | 0.0230 |
| Total | 45 | 180 | 225 | 300 | 300 | 450 | 1561939 | 1611196 | 2043557 | 2985448 | 2826442 | 2636437 |  |  |  |  |  |  |

● Line 153. I think it would be great to show the distribution of the titers of the 1500 participants rather than mentioning the percentages in the text or shown in a table. The distribution can be shown with a histogram with the titers on the x-axis.

**Response> Thank you for your valuable comments. As per your recommendation, we have added supplementary Figure 2 which shows the distribution of the IFA titers of the 1500 participants by region, please see supplementary Figure 2.**

**⇒ Supplementary Fig 2. Geographical distribution according to antibody titers of the 1500 participants by regions collected from the Korea National Health and Nutrition Examination Survey, 2014 to 2015. The location of the dot showing the antibody titers in each region was randomly assigned in each region by QGIS 3.26 program (**[**https://qgis.org/ko/site/**](https://qgis.org/ko/site/)**) using map obtained from GIS Developer site (http://www.gisdeveloper.co.kr/?p=2332).**

● Line 185 / Figure 3. I wonder whether the current representation is the best way. I am unsure what the message of the map is. Percentages are very hard to read. I would advise to use a choropleth map and choose one cut-off to be shown. This will probably underline the message that urban areas are less affected than rural areas.

**Response> Thank you for your valuable comments. As per your recommendation, we have prepared new Figure 3A which is a choropleth map using one cut-off.**

**⇒ Figure 3A. Choropleth map showing national SFTS seroprevalence (%) with IgG titer of ≥ 1:32. Map was obtained using “Editable South Korea Map Template For Presentation” (**[**https://www.slideegg.com/powerpoint/south-korea-map-powerpoint-templates**](https://www.slideegg.com/powerpoint/south-korea-map-powerpoint-templates)**).**

● Line 202 / Figure 4. I would focus less on finding a significant correlation and focus more on explaining / describing your data. Among those points is there any pattern in terms of rural areas vs urban areas? You could assess this by giving different colors to the points. Perhaps it reveals a certain pattern which tells you something about the surveillance of this disease.

**Response> Thank you for your valuable comments. First of all, there was no consistent pattern in terms of rural areas vs urban areas. However, it was observed as if there was a regionally consistent pattern between IFA positive rates (titer ≥ 1:32) and SFTS incidences per 100,000, and between IFA positive rates (titer ≥ 1:64) and SFTS incidences per 100,000, respectively. That is, the region Gangwon, Gyeongbuk, and Jeju had the highest SFTS incidence per 100,000 in 16 cities and provinces in Korea, and IFA IgG positive rate of ≥1:32 (%) and IFA IgG positive rate of ≥1:64 (%) were also the highest in these regions (Figure 3B). However, when we performed spearman correlation analysis to access the regional association between antibody positivity rates and SFTS incidence per 100,000, we only observed significant correlation between SFTS incidence per 100,000 and IgG seroprevalence with a titer ≥ 1:64 (p = 0.002) (Figure 4).**

**SFTS incidence per 100,000 in 16 cities and provinces in Korea, IFA IgG positive rate of ≥1:32 (%), and IFA IgG positive rate of ≥1:64 (%) in each region.**

| Region | SFTS Incidence per 100,000 | IFA IgG positive rate ≥1:32 (%) | IFA IgG positive rate ≥1:64(%) |
| --- | --- | --- | --- |
| Seoul | 0.05% | 3.05% | 1.69% |
| Busan | 0.00% | 4.76% | 2.38% |
| Daegu | 0.24% | 7.53% | 2.15% |
| Incheon | 0.14% | 7.45% | 2.13% |
| Gwangju | 0.00% | 1.85% | 0.00% |
| Daejeon | 0.13% | 3.70% | 1.85% |
| Ulsan | 0.17% | 3.03% | 3.03% |
| Gyeonggi | 0.12% | 2.59% | 1.72% |
| Gangwon | 1.23% | 8.00% | 6.00% |
| Chungbuk | 0.13% | 0.00% | 0.00% |
| Chungnam | 0.34% | 1.59% | 0.00% |
| Jeonbuk | 0.11% | 3.57% | 1.79% |
| Jeonnam | 0.52% | 0.00% | 0.00% |
| Gyeongbuk | 1.04% | 5.48% | 2.74% |
| Gyeongnam | 0.45% | 2.47% | 0.00% |
| Jeju | 2.66% | 6.25% | 6.25% |

● Line 202 / Table 2/3. Please provide a reason why you are looking at seropositivity at different cut-off values. To me your current approach is like P-value hacking, and it is probably better to stick to one cut-off.

**Response> In this study, we investigated SFTS seroprevalence using IFA IgG and IgM antibody titers ≥ 1:32 as positive cut-off values based on results from 15 health check-up participants. Moreover, we investigated SFTS seroprevalence rate when different cut-off values such as 1:32, 1:64, 1:128, and 1:256 were used.**

**In an ELISA for the diagnosis of SFTS, researchers generally use the mean + 3SD (standard deviation) of the negative control as a positive cutoff. However, in an SFTS IFA, researchers use different cut-off values as a criterion for judging SFTS positivity. According to report by Sun JM et al., in which they investigated SFTS seroprevalence in southeastern China and used 1:20 as an indicative of infection (Ref.1). According to report by Lee HJ et al., they use 1:80 dilution for the IFA as the last serial dilution that showed clearly discernible IFA results (Ref. 2).**

Ref. 1) Epidemiol. Infect. (2015), 143, 851–856. doi:10.1017/S0950268814001319. Seroprevalence of severe fever with thrombocytopenia syndrome virus in southeastern China and analysis of risk factors.

Ref. 2) [J Vet Sci.](https://www.ncbi.nlm.nih.gov/pmc/articles/PMC5366300/) 2017 Mar; 18(1): 33–38. doi: [10.4142/jvs.2017.18.1.33](https://doi.org/10.4142%2Fjvs.2017.18.1.33). A serological study of severe fever with thrombocytopenia syndrome using a virus neutralization test and competitive enzyme-linked immunosorbent assay

**Moreover, in our previous study (data not shown) investigating SFTS seroprevalence using 103 blood samples collected from Korean veterinary-hospital workers, the SFTS seroprevalence showed positivity rates of 3.9% (4/13), 2.9% (3/103), and 1.9% (2/103) according to an ELISA, IFA, and PRNT (plaque reduction neutralization antibody test), respectively. Antibody positivity was defined as follows: an IFA IgG titer of ≥1:32, ELISA IgG positivity (OD≥ mean + 3SD), and PRNT positivity (titer≥10). So to speak, SFTS seroprevalence determined by ELISA and IFA was similar. Moreover, using cut-off of ≥1:16 for IFA has a probability showing false-positive. Therefore, we used IgG and IgM antibody titers of ≥ 1:32 as positive cut-off value to determine SFTS seroprevalence rate, and compared the SFTS seroprevalence rate according to using different cut-off such as 1:32, 1:64, 1:128, and 1:256, respectively.**

● Line 277. You conclude that you observed a significant difference between SFTS and influenza vaccination, but for two cut-offs you did not observe this.

**Response> To avoid misunderstanding, we have added “with titers ≥ 1:128”.**

● Line 290. The manuscript would benefit from assessing how your estimate would change if your samples would be a better representation of the Korean population. With the Figure I suggested you to make, you’d have a good impression how representative your sample is in relation to age and region. Then you could elaborate how sure you are about your estimate of 3.7%, and in what direction you expect it go had you had a better sample. Or perhaps even better try to provide a weighted seroprevalence.
**Response> Thank you for your valuable comments. In this study, the proportion of participants in 16 cities and provinces was 0.0024~0.0053% of the population of each region (supplementary Table 1 and 2). Moreover, samples from Korea National Health and Nutrition Examination Survey could not be selected as the researchers wanted. The proportion of participants from urban (*dong*) and rural (*eup/myun*) areas were 81.2% (1218) and 18.8% (282), respectively. Population of the rural area was too small to be representative of rural area. Additional studies with a larger rural area population are needed.**

Minor points
● Line 63. To an international reader the term 16 cities and provinces does not provide any additional context. Please specify whether the sample is representative of the country or not.

**Response> Thank you for your valuable comments. As per your recommendation, we have changed it as follows.**

**⇒ This is the first study to investigate national SFTS seroprevalence across all 16 cities and provinces representing Korea; furthermore, our study will also provide useful guidelines for the development of preventive measures against SFTS.**

● Line 70: What does group 3 disease mean? Perhaps better to describe why it is categorized as a group 3 disease. Or describe to the reader why it matters.

**Response> In Korea, legal infectious diseases are divided into five groups (Group 1~5).**

**Group 3 infectious diseases are infectious diseases such as malaria, tuberculosis, scrub typhus, SFTS etc. that may prevail intermittently, requiring continuous monitoring of outbreaks and establishment of preventive measures.**

**As per your recommendation, we have changed it as follows.**

**⇒** **In Korea, among the five groups of legal infectious diseases, SFTS is a Group 3 infectious disease. Furthermore, since the first case was identified in 2013, its incidence has increased annually up to 2020 [3]. Group 3 infectious diseases include malaria, tuberculosis, scrub typhus, and SFTS. These diseases may prevail intermittently; therefore, continuous monitoring of outbreaks and the establishment of preventive measures are required (**[**https://www.kdca.go.kr/npt/biz/npp/portal/nppLwcrIcdMain.do**](https://www.kdca.go.kr/npt/biz/npp/portal/nppLwcrIcdMain.do)**).**

● Line 194. I think you can remove “data not shown” here as well as in line 177. Data is mentioned in text.

**Response> As per your recommendation, we have removed it.**

● Line 225 / Line 231. Please have a look at the titles of the tables. Title of table 2 is missing.

**Response> Thank you for your valuable comments. Now, you can see the title of Table 2.**

● Line 275. How related are SFTS and Influenza? It would be nice if this would be discussed in the discussion or introduction briefly.

**Response> Thank you for your valuable comments. SFTS and influenza itself does not seem to be related at all. In addition, we could not find any articles describing a relationship between SFTS seroprevalence and influenza vaccination. We’d like to suggest there is some chance of relationship between SFTS seroprevalence and biologic serologic false positive due to influenza vaccination.**

**To clarify it we have changed “This seropositivity may be related to influenza vaccination.” to “This SFTS seropositivity may be related to biological false positives due to influenza vaccination”.**

**Reviewer #2**

**Introduction**
● The introduction is too short and the similar work has already been done in some other countries, it could be great to develop the introduction with knowledge coming from other countries. There is even no information about the surrounding countries or even data in China, there have been several studies that have used the same study design as yours. Only from my point of view, the introduction should be rewritten.

**Response> Thank you for your valuable comments. As per your recommendation, we have added more references into “Introduction” of our revised manuscript as follows.**

**⇒ In China, systematic reviews and meta-analyses to evaluate STFS seroprevalence were investigated up to November 2016, and an overall pooled antibody positivity rate of 4.3% was reported [4]. In SFTSV antibody testing on 2,510 residents in Jiangsu Province, China, 0.44% of the residents tested positive [5]. However, the seropositivity rate for farmers in rural areas of the same province was 1.3%, which was three times higher than that of the general population [6, 7]. In addition, the seroprevalence of SFTSV infections was tested in 1,375 healthy persons from Penglai County, eastern China, and the serum of 3.85% of the general population tested positive for SFTSV antibodies [8]. In contrast, 7.2% of healthy residents in Zhejiang Province, China were positive for SFTSV IgG antibodies [9]. According to a report in 2018 that examined SFTS seroprevalence, 0.3% (2/646) of healthy individuals residing in Kagoshima, Japan, were positive for anti-SFTS antibodies. Moreover, 1,000 serum samples collected from general blood donors by the Japanese Red Cross Kyushu Block Blood Center were negative for anti-SFTSV antibodies, suggesting a low seroprevalence of SFTS antibodies in individuals living in endemic areas in Japan [10].**

**Our group previously assessed antibody titers according to regional and residential characteristics, tick contact history, and occupational and outdoor activity-related characteristics by investigating antibody positivity rates in residents of high-risk areas. Fifty (4.1%) of the 1,228 residents of rural areas exhibited an SFTS antibody titer of > 1:32, of which 23 (5.5%) in Boseong, 16 (4.0%) in Hapcheon, and 11 (2.7%) in Dangjin were seropositive for the SFTSV [11]. In addition, in a study conducted on 1,069 serum samples from patients who visited a tertiary hospital in southeastern Korea, 2.1% of the patients were positive for anti-SFTSV antibodies, of which 7.7% of patients from rural areas and 1.9% of patients from urban areas were shown to be seropositive [6].**

**Material and methods**
● **1. The field study needs to be better explained as to how the sample had been collected and for what purpose? During which months were the samples collected?**

**Response> The Korea National Health and Nutrition Examination Survey (KNHANES) is a national surveillance system that has been assessing the health and nutritional status of Koreans since 1998, and has been conducted by the Korea Disease Control and Prevention Agency (KDCA). In this study, we received stored serum samples collected for KNHANES to investigate nationwide SFTS antibody positivity rate. A total of 1,500 stored serum samples were received in two batches.**

**As per your recommendation, we have added following sentence to Materials and methods section.**

**⇒ We received 1,500 stored serum samples collected for KNHANES from the National Biobank of KDCA in two batches (800 samples for the first (March 30, 2018) and 700 samples for the second (April 06, 2018)), and stored them at -20 °C until use.**

● 2. An IFA image of a higher quality should be provided, also accompanied by a negative control image showing no staining.

**Response> Thank you for your valuable comments. As per your recommendation, we have added supplementary Figure 1 showing IFA images accompanied by a negative control image showing no staining as follows.**

**⇒ Supplementary Fig 1. Representative indirect immunofluorescence antibody assay results showing the presence of SFTS-specific IgG antibody using serum samples collected in KNHANES.**

**A. IFA image of positive control; B. IFA image of negative control; C. Negative control image showing no staining; D. No.149 serum of the 1st batch (positive, image using serum with 1:32 dilution); E. No.108 serum of the 2nd batch (positive, image using serum with 1:32 dilution); F. No.337 serum of the 2nd batch (negative, image using serum with 1:32 dilution).**

● 3. There needs to be a more detailed description of the IFA method. At what concentration did you use the secondary antibody?

**Response> We used 1:400 dilutions of FITC-conjugated anti-human IgG and IgM (MP Biomedicals) as the secondary antibodies, and this content was added to the revised MS.**

● 4. Statistics: A multivariate logistic might be better, as there are obviously collinearity between age and influenza vaccination. A trend analysis should be performed for the age comparison.
**Response> Response> We appreciate the reviewer' comments, and we agree that multivariate logistic regression is a better way to identify factors related to seropositivity. However, due to the small number of sample size** **with seropositivity, we could not conduct a multivariate analysis. Previous studies on sample size for logistic regression analysis recommended at least 20 events per variable (Austin & Steyerberg. Stat Methods Med Res. 2017; Bujang et al., Malays J Med Sci. 2018). We added this point as a limitation as follows in our discussion section.**

**⇒ Finally, due to the small number of seropositive samples, we could not conduct a multivariate analysis to identify factors related to seropositivity.**

**Results**
● 1. How about the occupation of the subjects, and is there any difference for the IgG titier regarding their occupation?

**Response> We divided the participants’ occupation into 4 groups, and analyzed SFTS seropositivity between 4 groups: (1) Office/service, (2) Agricultural/forestry/fisheries, (3) Production/technical, (4) Soldier (Table 3).**

**In detail, (1) Office/service (persons engaged in management, office work, service and sales; 556 participants; 4.3% seropositivity with titer≥1:32),**

**(2) Agricultural/forestry/fisheries (persons engaged in agriculture, forestry, fisheries; 80 participants; 2.5% seropositivity with titer≥1:32), (3) Production/technical (Physical labors engaged in equipment or machine operation, assembly workers, and simple labor workers; 294 participants; 3.4% seropositivity with titer≥1:32), (4) Soldier (2 participants; 0% seropositivity with titer≥1:32).**

**There was no significant difference between SFTS seroprevalence and occupational category. Please, see occupation in table 3.**

● 2. The IgG positive sample should be shown for their neutralizing antibody.

**Response> Thank you for your valuable comments. Unfortunately, there are currently no sera remaining, Government requested us to discard all the remain samples right after the end of the study. Therefore, PRNT(plaque reduction neutralization test) to measure SFTS neutralizing antibody is not possible at this time.**

● 3. Are there the simultaneously positive test from the same sample?

**Response> None of the 1500 participants tested positive for both IFA IgG and IgM at the same time.**

● 4. Lines 153: I can not understand the meaning of the sentences “The final antibody titer of the SFTS IFA IgM-positive sample was 1:64 (Table 1). Please rephrase.
**Response> As per your recommendation, we have rephrased it as follows.**

**⇒ The final antibody titer of one participant who tested positive for SFTS IFA IgM was 1:64.**

● 5. Is there any qPCR test for those IgM positive samples?

**Response> We performed real-time RT-PCR for IgM positive sample, but the result was negative.**

● 6. You might need to list the months of the positive samples and compared with the negative ones.

**Response> Unfortunately, when we received 1,500 stored serum samples collected for KNHANES from the National Biobank of KDCA, we didn’t receive the information related with sample collection months of participants from KNHANES, so additional analysis is not possible. We only received the information related with collection year.**

**Discussion:**
● You need to comment on how comorbidities could have contributed to the IgG positive detection.

**Response> We don’t think comorbidities itself contributed to the IgG positive. In our previous study the older the population is the more seropositive for SFTSV. Older age person tend to have more comorbidities. In addition, Government recommend influenza vaccination in older age group and comorbidities group, therefore higher chance of biological false positive.**

● The refences are not properly cited, for example, the discovery of SFTSV in China should be the NEJM paper, instead of the ref 2 (Kim KH, Yi J, Kim G, Choi SJ, Jun KI, Kim NH, et al. Severe fever with 307 thrombocytopenia syndrome, South Korea, 2012. Emerg Infect Dis. 2013;19(11):1892-4).

**Response> Thank you for your valuable comments. We have changed the reference as per your suggestion.**

● The identification of risk factors of seroprevalence is not refenced adequately, I would add reference in China (PMC4350533, Hu C et al；Ye X et al, PMC8600349) for the comparison with the risk factor related to antibody production in an endemic Region.
**Response> Thank you for your valuable comments. We have added two references on our revised manuscript. Moreover, we have added following sentences into Discussion section.**
**⇒ According to a study on SFTS seroprevalence in Xinyang—the most severe SFTS-endemic region of China—the seropositive rate was 6.59%. In addition, the SFTS seroprevalence was higher in individuals who lived in rural areas and in people that were sampled in post-endemic seasons, suggesting that the seroprevalence of SFTSV-specific IgG antibodies was significantly correlated with the reported incidence rate and temporal and geographic level [14].**

**⇒ In a cohort study of a healthy population in a highly endemic region of China, the overall seropositive rates were 11.9% (IgG antibody) and 6.8% (neutralizing antibody), respectively. Moreover, it was reported that anti-SFTSV IgG seropositivity was significantly related to older age (≥70 years), recent contact with cats, and working in tea gardens [15].**
