# Supplementary material for: Seroprevalence of severe fever with thrombocytopenia syndrome using specimens from the Korea National Health & Nutrition Examination Survey
Source: PLoS Negl Trop Dis. 2023 Mar 22;17(3):e0011097. doi: 10.1371/journal.pntd.0011097 (PMC10032665; doi:10.1371/journal.pntd.0011097)
Supplement: Attachment — Submitted filename: Response letter_3rd revision.docx [file pntd.0011097.s006.docx]

**To the Editor of PLOS Neglected Tropical Diseases**

**Re: Manuscript ID: PNTD-D-22-00247R2
Seroprevalence of severe fever with thrombocytopenia syndrome using specimens from the Korea National Health & Nutrition Examination Survey**

Dear Editor,

First, I would like to thank you on behalf of all of my co-authors for reviewing our manuscript. Sorry for the late reply due to late email confirmation.

Our responses to the comments made by the reviewers are included below in bold.

Thank you for considering our manuscript, and we hope that this draft of our manuscript will be of interest to the readers of “PLOS Neglected Tropical Diseases*”*.

**Reviewer(s)' Comments to Author:**

1) Thank you for including your ethics statement: " This study was approved by the Institutional Review Board (IRB) of Chosun University Hospital (IRB No. 2017-12-014). Written informed consent was obtained from all participants for the use of blood samples in KNHANES. Written informed consent was obtained from the parent/guardian/head of household in case of participants less than 18 years old" . Please state the full name of KNHANES.

**Response> Thank you for your valuable comments. As per your recommendation, we have changed “KNHANES” to “Korea National Health and Nutrition Examination Survey”.**

**Re: Manuscript ID: PNTD-D-22-00247R1
Seroprevalence of severe fever with thrombocytopenia syndrome using specimens from the Korea National Health & Nutrition Examination Survey**

Dear Editor,

First, I would like to thank you on behalf of all of my co-authors for reviewing our manuscript.

Our responses to the comments made by the reviewers are included below in bold.

Thank you for considering our manuscript, and we hope that this draft of our manuscript will be of interest to the readers of “PLOS Neglected Tropical Diseases*”*.

**Reviewer(s)' Comments to Author:**

● Reviewer #2: The authors had addressed most of the queries, however, some information should also be added to the text.
The authors should use appropriate statistic term, e.g.,Line 146, The chi-squared test or Fisher’s exact test was to assess the difference not the correlation between the investigated risk factors and SFTS.

**Response> Thank you for your valuable comments. As per your recommendation, we have changed “correlation” to “difference”.**

● Line 259, In addition, there was a significant difference between SFTS seroprevalence with titers ≥ 1:128 and influenza vaccination---please rephrase/

**Response> As per your recommendation, we have rephrased it as follows.**

**⇒ In addition, there was a significant correlation between SFTS seroprevalence with titers ≥ 1:128 and influenza vaccination.**

● The chi-squared should be Chi-square

**Response> We have changed “chi-squared test” to “Chi-square test”.**

● Distribution of Sample Characteristics should be Sample Characteristics or Distribution of Sample

**Response> We have changed “Distribution of Sample Characteristics” to “Sample Distribution”.**
